# Enhancing strategic defensive positioning and performance in the outfield

**DOI:** 10.1007/s10109-021-00367-1

**Published:** 2022-01-10

**Authors:** Alan T. Murray, Antonio Ortiz, Seonga Cho

**Affiliations:** Department of Geography, University of California at Santa Barbara, Santa Barbara, CA 93106 USA

**Keywords:** Location modeling, Spatial optimization, Sports analytics, C61, L83, O21

## Abstract

Over the past 20 years, professional and collegiate baseball has undergone a transformation, with statistics and analytics increasingly factoring into most of the decisions being made on the field. One particular example of the increased role of analytics is in the positioning of outfielders, who are tasked with tracking down balls hit to the outfield to record outs and minimize potential offensive damage. This paper explores the potential of location analytics to enhance the strategic positioning of players, enabling improved response and performance. We implement a location optimization model to analyze collegiate ball-tracking data, seeking outfielder locations that simultaneously minimize the average distance to a batted ball and maximize the weighted importance of batted ball coverage within a response standard. Trade-off outfielder configurations are compared to observed fielder positioning, finding that location models and spatial optimization can lead to performance improvements ranging from 1 to 3%, offering a significant strategic advantage over the course of a season.

## Introduction

Marketing and popular media often refer to baseball as America’s pastime, suggesting that it is the most popular sport in America. Whether this is true is open for debate, but clearly it is a broadly popular sport with a huge following. Baseball (and softball) shares similarities to cricket and rounders, among other games, and as a result, there has been considerable speculation about its origins. But there is no denying its popularity worldwide, either in terms of participants or spectators. Some 25 million athletes play the sport baseball or softball (Nuckols 2019), with estimates of more than 500 million fans worldwide. Countries where baseball is particularly popular include the USA, Japan, Puerto Rico, Dominican Republic, Cuba and Canada, and it was among the first organized sports to return amid the COVID-19 pandemic in places like Taiwan and South Korea.

Baseball pits two teams against each other and involves a pitched ball by a player on one team (from the pitcher’s mound) that an opposing player attempts to hit with a bat standing in the batter’s box at home plate. Upon a batted ball in the field of play, the batter tries to advance to one or more bases without being tagged out. Advancing across all three bases (first, second and third) and returning to home plate records a run. The team that scores the most runs wins. There are many rules and requirements for play, but the essence of the game is that a batted ball in the area of play entitles the batter to advance bases. A batted ball that is caught by the opposing team registers an out. Otherwise, the batter may run to consecutive bases as long as they are not tagged out by an opposing player that has possession of the ball. Standing on a base without being tagged en route constitutes safe advance of the bases. Of course, there are many more rules and nuances to the game. Indeed, the rule book for Major League Baseball (2019) is more than 140 pages, plus another 30 pages for terms, definitions, specifications, etc.

Of particular interest here is that the fielding team must position nine players in order to catch or field a batted ball, seeking an out (either by catching the ball before it touches the ground or by tagging a batter before they reach a base). The pitcher is required to be on the pitcher’s mound, and the catcher must be positioned behind home plate, but the remaining seven players are permitted to be anywhere in the playing area. Historical convention is that four of the seven players are distributed in the infield to help guard/cover bases and three are positioned in the outfield. Fielder location is clearly important strategically as this will enable quicker response to a batted ball. There have been circumstances where four or more outfielders have been used (Miller 2017), creating speculation and discussion about the effectiveness of traditional conventions to fielder placement.

Statistics and analytics have played an increasingly important role in baseball and other sports, enabling insights and gains in predictive capabilities. Noteworthy in baseball is sabermetrics, advanced statistical and empirical analysis. Sabermetrics contributed to the success of the Oakland A’s in the late 1990s and early 2000s and were popularized in the Moneyball book by Lewis (2003) as well as the subsequent feature film starring Brad Pitt and Jonah Hill based on this book. Of significance is that much data are collected and tracked in professional and collegiate baseball associated with pitchers, batters, fielders, etc. Why? A competitive advantage may be gained through the detection of trends and insight that are revealed in data. Further, better financial investment decision making may be possible. It should come as no surprise then that modern technology is heavily relied upon in data collection and tracking efforts. The TrackMan radar system is particularly common at collegiate and professional ballparks, enabling instantaneous information on ball velocity as well as bearing, distance and location information. While statistical and empirical analysis of such data is widespread, the potential to exploit this information in strategic ways continues to be of great interest.

This paper explores the potential of location analytics to support enhanced response and performance through the strategic positioning of outfielders. While much has been made of recording extra outs with the implementation of infield shifts (Lewis and Bailey 2015), there has been less attention to the strategic positioning of outfielders. This is a major oversight as balls hit to the outfield generally have more potential to produce runs. Thus, increasing the chance to record out or improving response time in the outfield is particularly valuable. Location analytics offer the potential to inform and enhance outfielder positioning, moving beyond the subjective and arbitrary approaches that have historically been relied upon and doing so based on objective data-driven methods. The next section reviews relevant background literature. This is followed by introduced analytical methods, in particular a multi-objective spatial optimization model. The positioning of fielders is evaluated using recorded batted ball data for a university baseball team. The paper ends with discussion and concluding comments associated with the enhanced strategic placement of fielders.

## Background

Sports are woven into the fabric of modern day life in most countries. Many may think of community recreation teams where all players participate. And no doubt sports are popular for exercise and activity as well as providing life lessons about working as a team. Youth and adult leagues along these lines are commonplace. However, professional leagues demonstrate that many sports are anything but recreation. Baseball certainly reflects this, with extensive interest and participation at the youth level, through organizations like Little League Baseball and Softball (with different age divisions such as Tee Ball, Minor League, Major Division, Intermediate, Junior League and Senior League), up through high school sponsored programs, college/university teams and professional teams (which have an extensive Minor League or farm system that develops players). Even the most basic level of organization involves administrative oversight, equipment, coaches, fields, participants, player development, travel, etc. There are real costs associated with each of these, making it an industry. Baseball and most popular sports are indeed big business.

Major League Baseball is a multi-billion dollar industry, reflecting the serious side of this sport and its broad community and regional economic implications. Much is invested in monitoring and analysis of individual player and team performance because more successful teams garner more spectator interest. To this end, extensive statistical and other analysis has emerged, relying on a wealth of associated data. Mentioned previously was that sabermetrics has proven to be advantageous in many ways, aiding in the management of team composition, particularly rosters, lineups and development. All teams now invest in the capacity to quantify and analyze performance. Of course, this is not limited to baseball either. Goldsberry (2019) discusses the use of analytics, including cartographic and geographic information system-based approaches, to better understand shooting performance in the NBA (National Basketball Association). The point is simply that analytics through mapping, statistical summary and more sophisticated approaches are now somewhat standard, at least for professional and collegiate sports of all kinds.

Analytics in baseball range from measures and metrics to formal statistical models and tests (Koseler and Stephan 2017; Marchi et al. 2018; Elitzur 2020; Ratten and Hayduk 2020). Point and heat maps to measure batter decision making are common, as is the automation of pitch location mapping to determine balls and strikes. Precise pitch tracking and spin data are used for player development. Hitter performance estimation and prediction is regularly used along with fielder range to objectively evaluate defensive ability, and many professional teams now rely on predictive metrics to narrow down future contributions to the amount of additional runs or wins a particular player is expected to provide. Modeling also supports salary and arbitration along business analytics lines, as well as long-term play valuation.

This new era of baseball analytics is a by-product of a plethora of new technologies providing an increasing amount of data. It began with PITCHf/x camera systems, which tracked the location of a pitch, and eventually expanded to TrackMan, a military grade radar system that provides ball-tracking data such as velocity, spin rate, positions and angles of flight. Major League Baseball teams now use Hawkeye, a camera-based system that tracks player movement as well as the ball and bat.

The increase in available data has led to a demand for much more in depth study of the positioning of defenders. Whereas Major League Baseball teams previously played “straight-up,” or with a symmetrical outfield alignment, recent years have seen more shifting, likely based on both statistical positioning and in-game intuitive adjustments. At the collegiate level, less data are available, leading to more teams continuing to implement the traditional approach.

Gerchak (1994) and Wright (2009) review the use of operations research or optimization in sports. Optimization is an analytic approach where decisions are to be made, with the problem often characterized mathematically as a function(s) of decision variables. Three basic categories of usage can be identified: strategy, scheduling and forecasting. Strategies examined in baseball include batting order, use of a pinch runner and general substitution, but also training components. Schedule development in baseball includes things like umpire assignments noted in Evans (1988), Trick et al. (2012) and De Oliveira et al. (2016) as well as game locations. General work on team and conference scheduling can be found in Nemhauser and Trick (1998) and Duran et al. (2007). Forecasting has to do with prediction of outcome, etc. Thinking about optimization in these contexts, the usage of optimization is indeed broad. With respect to scheduling, the idea is to identify a schedule of games to be played over a season involving many teams. The hope is that schedules for each team can be developed that are fair and equitable, but also perhaps involve the least amount of travel and keep costs to a minimum (see Van Bulck and Goossens 2020 for a general discussion). The decisions therefore are which team is to play other teams and where and when each game is to be played. Constraints are often associated with limiting too much travel for any team in consecutive weeks and ensuring a minimum number of home games.

Until recently, there was very little positioning research in the public domain, as the ball flight data were not readily available outside of Major League Baseball organizations. With the publishing of Baseball Savant by Major League Baseball, the public now has access to a wealth of ball-tracking data. Preliminary work on strategic fielder positioning is that of Easton and Becker (2017), formulating an integer program based on the probability of a hit along with constraints that enforce subjective “reasonable positioning.” Gerlica et al (2020) describe a clustering-based approach for outfielder shifting focused on access. Finally, an iterative looping heuristic is mentioned in Montes (2021) for positioning outfielders with respect optimizing to coverage. No specific details are given about the approach, nor is it formalized in any way that could be replicated. In contrast to the above work, our approach is to optimize defensive positioning at the collegiate level with an individual set of TrackMan data, which unlike Savant data is more raw and simply provides physical descriptions of a ball in flight.

An open research question is whether location analytics can further complement existing approaches used in baseball, and other sports. For example, location models are optimization problems involving siting decisions, usually a service facility of some sort. A review of the many different types of location models can be found in Church and Murray (2009). More generally, spatial optimization extends optimization to effectively any problem context involving geographic space. An overview of spatial optimization can be found in Tong and Murray (2012). Explored in this research is whether location modeling and spatial optimization can serve as an advanced sporting analytic approach in baseball. In particular, the positioning of fielders is of interest, taking into account both access and coverage issues simultaneously.

## Methods

Spatial analytics can be broadly characterized as any and all approaches that provide insights regarding the spatiotemporal distribution of geographic phenomena or facilitate associated decision making. Accordingly, such analytics could be descriptive or prescriptive, a distinction discussed in Church and Murray (2009). Often relied upon approaches include exploratory spatial data analysis, geographic information systems, measures, metrics, statistics, optimization, geosimulation, etc. (Murray 2017). Of interest in this research is optimization, and location models in particular. Spatial optimization and location modeling are often used to understand the performance of a spatial configuration or to design an ideal spatial configuration of a service system. This has great potential in the context of baseball. The service system can be conceptualized as the configuration of fielders, defending against hits. Description or prescription consists of one or more utility functions that reflect service quality. Associated with baseball, service quality for defensive purposes is invariably the ability of players to react and field a batted ball. There may also be constraining factors, such as the number of fielders to position. Locational decisions to be made revolve around the placement of players in order to defend the field of play.

As mentioned above, location modeling is a broad area of research and application, as detailed in Church and Murray (2009). To address issues of player positioning in baseball, two categories of location models are worth noting. The first is that of ReVelle and Swain (1970) who formulated the p-median problem given the description offered in Hakimi (1964) (see also Peeters and Thomas 2000). This optimization model involves location decision making through the use of variables to represent where to site facilities as well as the demand that they serve. This is one of the so-called location–allocation models, with an objective of minimizing the average distance of service provided to demand. Why is this of significance here? The answer is that good fielder positioning is predicated on the ability to respond to a batted ball, so minimizing average distance is critical for securing the ball in order to prevent additional run(s) from scoring. A second location model to mention is that of Church and ReVelle (1974), referred to as the maximal covering location problem. This optimization model seeks the configuration of facilities that is able to serve, or cover, the most demand within a maximum service time or travel distance standard (see Murray and O’Kelly 2002, Matisziw and Murray 2009; Chen et al. 2021). How is coverage relevant here? A batted ball that can be caught by a fielder before it touches the ground is an out. Thus, the range that a fielder is able to travel in order to catch a fly ball reflects the very essence of coverage.

Both location modeling approaches, the p-median and maximal covering, are important in the context of baseball. Ideally, one would be particularly interested in an approach that integrates the two. Church et al. (1991) and Pirkul and Schilling (1991) investigated the simultaneous use of both modeling approaches, though in a limited manner. In what follows below, an integrated location model is introduced. This optimization model shares obvious similarities with the work of Church et al. (1991) and Pirkul and Schilling (1991), but there are technical differences that are significant in a number of ways.

Consider the following notation:

$i =$ index of demand units (batted ball locations) (entire set $I$),

$j =$ index of potential fielder positions (entire set $J$),

$a_{i} =$ value / intensity of batted balls in unit $i$,

$d_{ij} =$ distance from position $j$ to unit $i$,

$N_{i} =$ set of positions that can field a batted ball to unit $i$,

$p =$ number of fielders to position,

$$X_{j} = \left\{ \begin{gathered} 1\quad {\text{if a fielder is placed at position }}j \hfill \\ 0\quad {\text{otherwise}} \hfill \\ \end{gathered} \right.$$,

$$Z_{{ij}} = \left\{ \begin{gathered} 1\quad {\text{if fielder at position }}j{\text{ assigned to catch a batted ball to unit }}i \hfill \\ 0\quad {\text{otherwise}} \hfill \\ \end{gathered} \right.$$.

The notation reflects important problem nuances. First, it is assumed that a finite number of potential locations to position players have been identified. This is the index $j$. With this, it is possible to conceive of the decision to be made for each potential location. Is a player positioned at $j$ or not? This is the intent and function of variable $X_{j}$, with binary options reflecting a yes or no decision at each possible position $j$. A second problem nuance is associated with observed batted balls. This is done using the index $i$. The batted ball locations necessitate an assignment or allocation of a fielder to respond. The response is therefore tracked using the decision variable $Z_{ij}$. With knowledge about position as well as player response, it is possible to structure performance measures associated with service quality. This can be done for any spatial configuration or pattern of defensive player positioning, enabling comparative evaluation. The integrated location model to reflect the primary goals and objectives in fielder positioning is as follows:

1 $$Maximize\quad \mathop \sum \limits_{i} \mathop \sum \limits_{{j \in N_{i} }} a_{i} Z_{ij}$$

2 $$Minimize\quad \mathop \sum \limits_{i} \mathop \sum \limits_{j} a_{i} d_{ij} Z_{ij}$$

3 $$Subject \, to\quad \mathop \sum \limits_{j} Z_{ij} = 1 \;\; \forall i$$

4 $$Z_{ij} \le X_{j} \;\; \forall i,j$$

5 $$\mathop \sum \limits_{j} X_{j} = p$$

6 $$X_{j} = \left\{ {0,1} \right\} \;\; \forall j$$

7 $$Z_{ij} = \left\{ {0,1} \right\} \;\; \forall i,j$$

This is a multi-objective spatial optimization model. Objective (1) seeks to maximize the total weighted demand (batted balls) covered. Objective (2) is oriented to minimize the average weighted distance to demand (batted balls). Constraints (3) indicate that each demand (batted ball) must be assigned to a fielder. Constraints (4) prevent assignments to only selected fielder locations. Constraint (5) specifies the number of fielders to be located. Constraints (6) and (7) impose binary restrictions on decision variables.

The optimization model incorporates the decisions to be made about player position in the context of two service quality measures, coverage within the standard and average response. These are the objective functions, (1) and (2), respectively. There is considerable interest and theory associated with the physics of a batted ball (Bahill 2019) as well as fielding ability. In this research, it is assumed that the range to catch a batted ball in the outfield is reasonably represented by the set $N_{i}$, potentially defined in a regular manner (e.g., $N_{i} = \left\{ {j{|}d_{ij} \le S} \right\}$, where $S$ is a maximum distance) or irregular in shape (see Axisa 2014). The regular case, of course, corresponds to a circle of radius $S$, where backward, forward and sideways response from an established location is equivalent. The irregular situation would reflect increased or decreased response range in one or more directions. Given this, objective (1) is a measure of expected fielder response to catch a batted ball before it touches the ground, based on the selected fielder locations. Objective (2) measures total weighted assignment distance. Average distance is the result of standardization of objective (2) by total weight, or rather total value of batted balls, as follows:

8 $$\frac{{\mathop \sum \nolimits_{i} \mathop \sum \nolimits_{j} a_{i} d_{ij} Z_{ij} }}{{\mathop \sum \nolimits_{i} a_{i} }}$$

Since the denominator in (8) is a constant, unchanged by decision variables, then the use of (2) or (8) is equivalent from an optimization perspective. For these objective functions to accurately measure or quantify service quality, constraints are needed. The allocation of service to each batted ball is reflected in constraints (3) and enables total weighted distance to be computed across all batted balls. This indicates that each batted ball must be fielded. Of course, allocation is only possible for the locations of a player, which is why constraints (4) are necessary. If location $j$ has been selected for a fielder, then $X_{j} = 1$. This would imply in (4) that $Z_{ij} \le 1$. Thus, $Z_{ij} = 1$ or $Z_{ij} = 0$ is possible. Alternatively, if location $j$ is not selected, then $X_{j} = 0$. This would imply in (4) that $Z_{ij} \le 0$. Thus, $Z_{ij} = 0$ is the only possibility in this case. The number of players to position is reflected in constraints (5), where the parameter $p$ is associated with the number that will be positioned. This is known and specified in advance of applying the optimization model. Collectively, models (1)–(7) stipulate performance measures to be optimized while ensuring that they logically result from the fielder location decision making process.

As with any optimization model, formulation and specification is the first step. One then needs the associated input data. In this case, one assumes that we have batted ball locations (index $i$) as well as potential player locations (index $j$). An interesting feature of this model is that the coefficient $a_{i}$ can be used to reflect that some batted balls may be more significant than others, with higher values indicating more importance. With these components, distance and coverage can be derived. Solving the resulting model is the next challenge. For any optimization model, there are two potential methods of solution, exact and heuristic. An exact approach is one where there is a guarantee that an optimal solution is identified upon successful termination of the approach. Alternatively, a heuristic is an ad hoc approach to identify a solution, often feasible with respect to constraints, but there is no ability to verify or prove solution quality. Model characteristics and problem application size often dictate whether an exact or heuristic method is used for solution. However, there does exist commercial and open-source software as well as specialized algorithms for problem solution, for both exact and heuristic methods.

Another complicating feature of the detailed location model, (1)–(7), is the presence of multiple objectives. Specialized techniques are necessary to deal with more than one objective. The reason is that a range of optimal solutions are possible, each reflecting a particular relative importance for the competing objectives. This is known as the Pareto frontier, defined by the set of so-called non-dominated solutions. A solution that is *non-dominated* is feasible, satisfying all constraints (3)–(7) in this case, with no improvement possible for one objective, (1), without degradation of the other objective, (2) (also referred to as Pareto optimal, Pareto efficient or non-inferior). The goal then is to identify tradeoff solutions for subsequent evaluation, so that decision making can occur that takes this into account. For exact solution of linear–integer models like that formulated here, two primary approaches to identify non-dominated tradeoff solutions are the weighting method and the constraint method (see Cohon 1978).

## Data

**Fig. 1:**
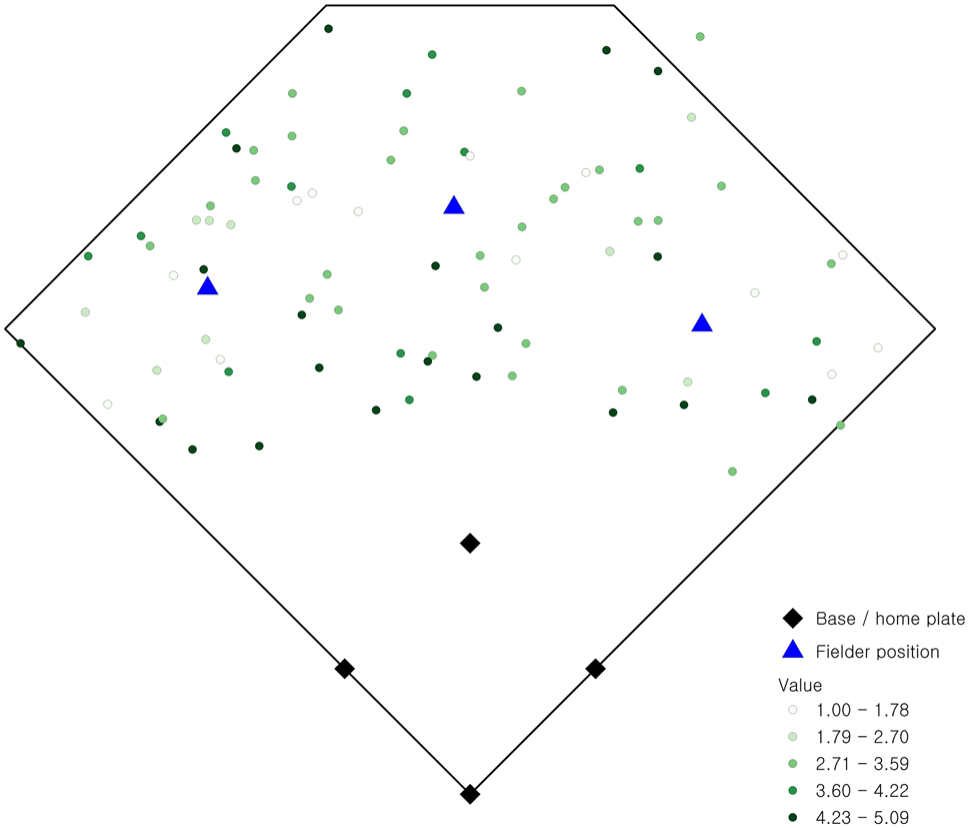
Spatial distribution of observed batted balls (and expected value)

Observed batted balls during college baseball games at the University of California at Santa Barbara are utilized in this research. Data for batted balls were obtained using the TrackMan radar system installed at Caesar Uyesaka Stadium during the 2018–2019 season for a number of different scenarios, including right- and left-handed pitchers as well as right- and left-handed batters. The field is shown in Fig. 1, with the distance along the foul lines (left field and right field) measuring 320 ft. and the furthest distances in center field measuring 390 ft. Other field dimensions are consistent with National Collegiate Athletic Association (NCAA) (and Major League Baseball, MLB) regulations, e.g., 90 ft. from home plate and first base, 90 ft. from home plate and third base, 90 ft. between consecutive bases, 60.5 ft. between home plate and the pitcher’s rubber, etc.

Included in this analysis are 85 batted balls by right-handed batters against a left-handed pitcher traveling over 200 ft. with at least 1.5 s of hang time. This represents the demand set, $I$. As discussed later in the paper, other types of batted balls and focused analysis based on pitcher and batter characteristics, such as a left-handed batter against a right-handed pitcher, could be carried out without any loss of generality in the methods and approach detailed. The data for each batted ball include velocity off the bat as well as angle (90° is a pop-up and − 90° is directly into the ground). These measurements are commonly referred to as exit velocity and launch angle and can provide a considerable amount of information about the potential run value of a batted ball. A random forest model using the above two metrics as predictors and the MLB run value of a given event type (e.g., single, double, flyout, etc.) were used to estimate the potential offensive value of a batted ball, $a_{i}$. This means that $a_{i} = f\left( {\text{exit velocity, launch angle, run value}} \right)$, where $f\left( {\cdot} \right)$ is a function. These values range from 1.0 to 5.090 (e.g., $a_{i} \in \left[ {1.0, 5.090} \right]$), with a mean of 3.306.

A discrete representation of the field of play was extracted for illustrative purposes as a regular collection of points spaced every 6 ft. The result is 2,977 points that serve as possible fielder positions, $$J$$. Euclidean distance is assumed, e.g., $$d_{ij} = \left[ {\left( {\phi_{i} - \hat{\phi }_{j} } \right)^{2} + \left( {\lambda_{i} - \hat{\lambda }_{j} } \right)^{2} } \right]^{{{\raise0.7ex\hbox{$1$} \!\mathord{\left/ {\vphantom {1 2}}\right.\kern-\nulldelimiterspace} \!\lower0.7ex\hbox{$2$}}}} ,$$ where $$\left( {\phi_{i} ,\lambda_{i} } \right)$$ are the Cartesian coordinates of demand $$i$$ and $$\left( {\hat{\phi }_{j} ,\hat{\lambda }_{j} } \right)$$ are the coordinates of potential fielder location $$j$$. The outfielder range is assumed to be 90 ft. given response possible within 4 s. This means that $$N_{i} = \left\{ {j{|}d_{ij} \le S} \right\}$$ for each demand, where $$S = 90$$. Other factors, such as different response capabilities of outfielders, field characteristics, etc., were not explicitly addressed in this preliminary research, but the modeling approach is capable of addressing regular or irregular coverage ranges.

## Strategic analysis

Assessment of fielder locations serves as a beginning point for this analysis. Figure 1 indicates the observed outfielder positions in defense of the observed batted balls. The relative value of each batted ball is also depicted in Fig. 1, with darker shades indicating a higher relative value. The observed fielder locations can be assessed with respect to the stated positioning objectives, (1) and (2). The total weighted value of batted ball coverage (1) in this case is 241.02, which is 85.78% of the total possible (280.98). The total weighted distance (2) for this configuration of fielders is 18,567.22, which corresponds to an average distance of 66.08 ft. (e.g., 18,567.22 ÷ 280.98) for the closest fielder to each observed batted ball.

Exploring whether measureable improvements are possible by changing fielder locations is investigated using spatial optimization models (1)–(7). This assessment is carried out using the constraint method to identify non-dominated solutions. Specifically, the coverage objective (1) is moved to a constraint requiring a specific total demand to be covered. This total is incrementally changed (0.01 demand units), and the problem repeatedly re-solved for select intervals beginning with total coverage of 213 up to 250. Xpress (version 8.8) was used to optimally solve all associated problem instances on a Windows 10 AMD Ryzen CPU 3900X (4.6 GHz) with 96 GB RAM desktop computer. In total, 501 problems were solved using the constraint method, requiring 69.09 s of computing time on average.

**Fig. 2:**
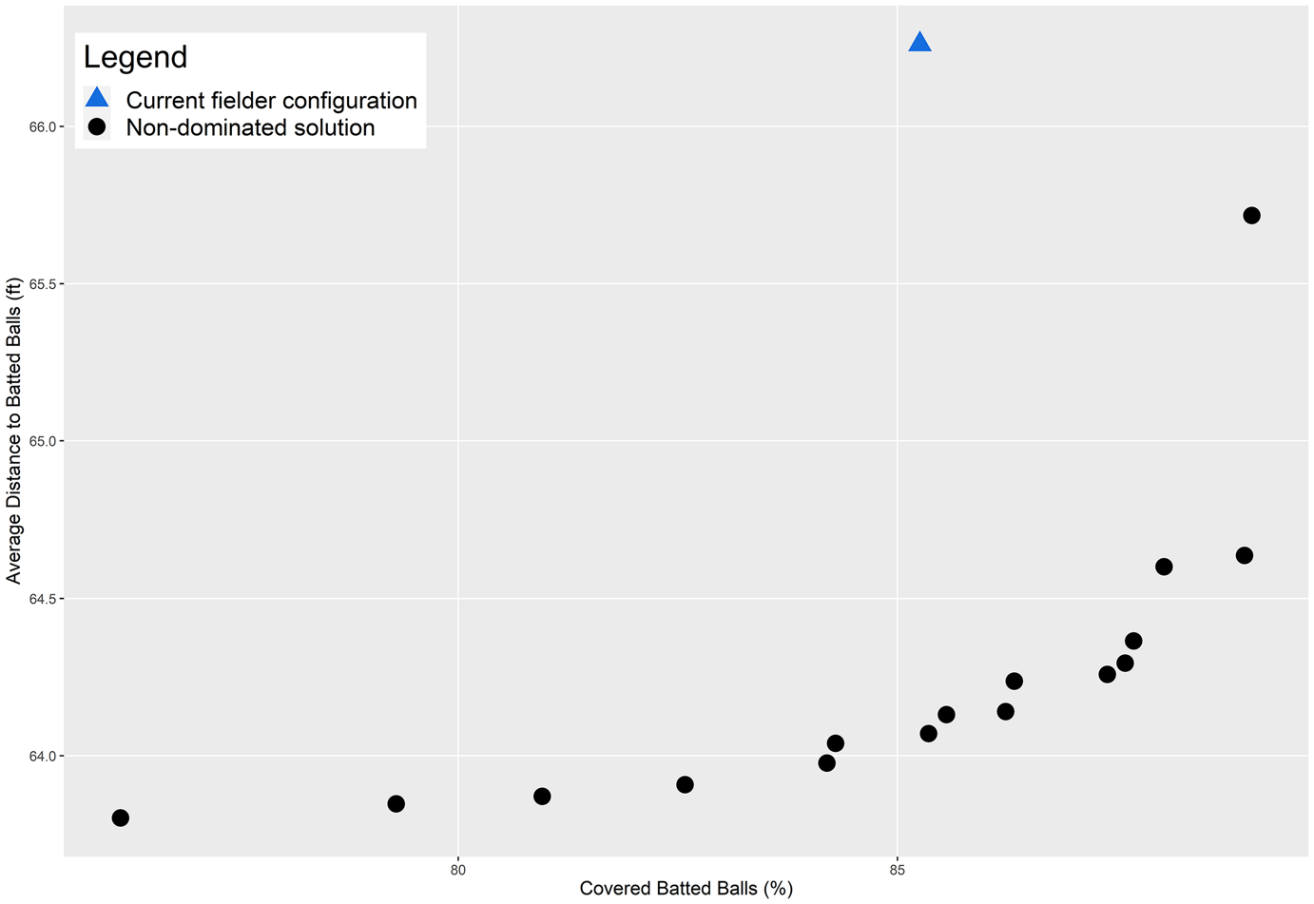
Coverage and average distance tradeoff solutions

**Fig. 3:**
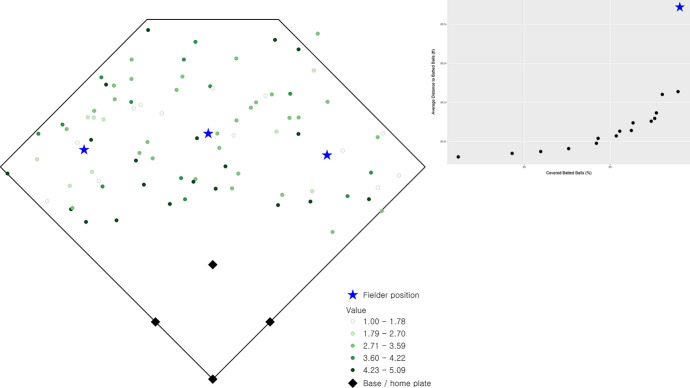
Fielder configuration favoring coverage objective, (1)

**Fig. 4:**
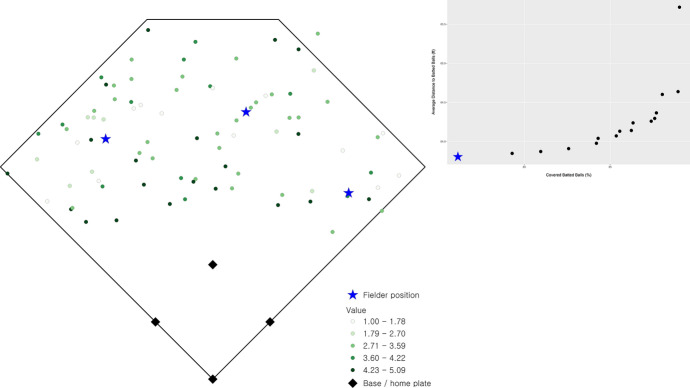
Fielder configuration favoring distance objective, (2)

**Fig. 5:**
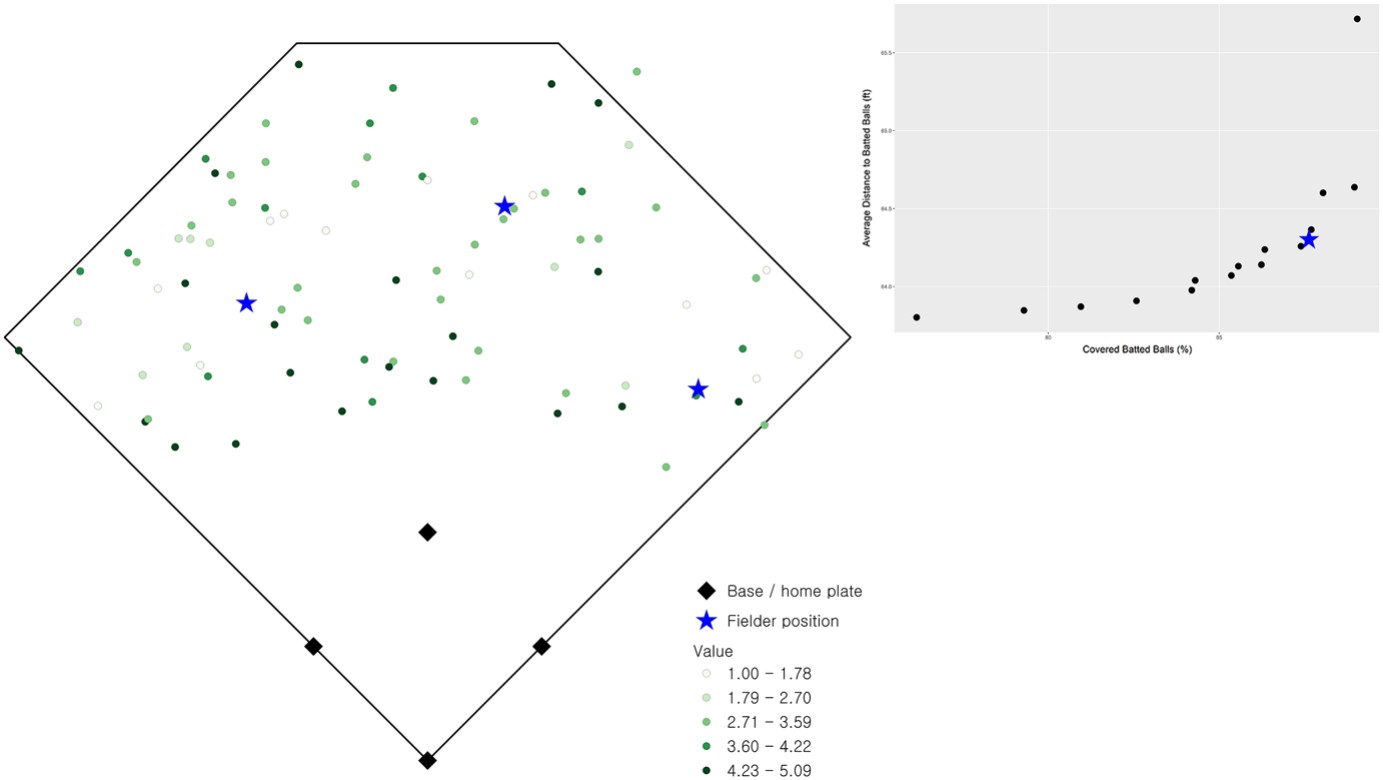
Fielder configuration compromise between objectives (1) and (2)

**Fig. 6:**
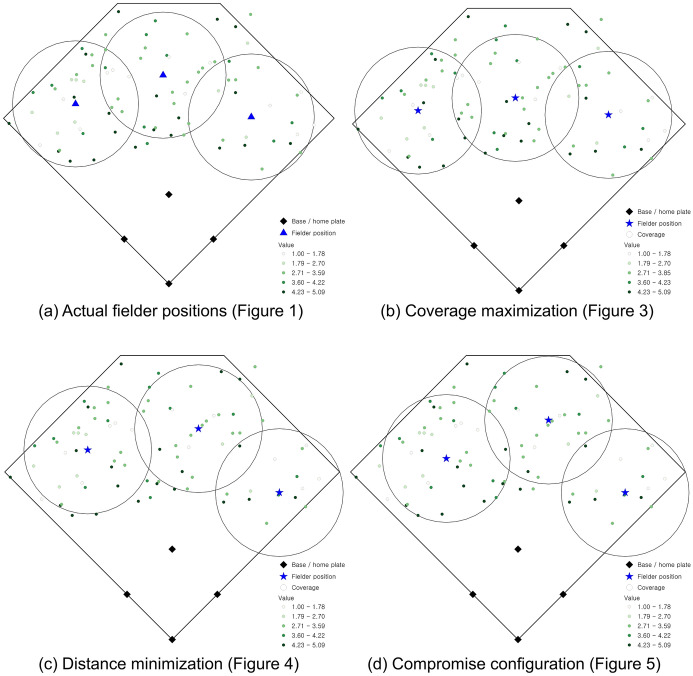
Fielder configurations with coverage range included

There are 16 non-dominated solutions identified, representing the range of optimal solutions that are efficient fielder positions relative to the stipulated objective criteria. These solutions are summarized in Fig. 2 as a percentage of total demand covered (x-axis) and average distance (y-axis), which is a simple standardization of the objectives (1) and (2), respectively. The two extreme solutions in Fig. 2 are worth explaining. One involves coverage of 89.04% of total demand giving an average access distance to a batted ball of 65.72 ft. The spatial configuration of outfielders in this case is shown in Fig. 3 (the inset highlights the corresponding tradeoff of non-dominated solutions given in Fig. 2). In essence, this configuration gives all emphasis to the coverage objective, (1). The other extreme covers 76.15% of demand with an average distance of 63.80 ft. The spatial configuration of outfielders in this case is shown in Fig. 4 (again, inset highlights this tradeoff solution), with the emphasis on average distance associated with objective (2). Both extremes establish performance bounds for tradeoff solutions, where coverage ranges from 76.15% to 89.04% and average distance spans 63.80 to 65.72 ft. All other depicted solutions in Fig. 2 represent tradeoff scenarios, where varying levels of emphasis are given to both objectives simultaneously. One such tradeoff solution is shown in Fig. 5 (inset highlights tradeoff), where coverage within the standard is achieved for 87.59% of total demand and the associated average distance to a batted ball is 64.29 ft. The coverage range for these different solutions is included in Fig. 6 in order to facilitate comparative assessment.

Returning to the actual fielder positions in Fig. 1 (coverage of 85.78% and average distance to observed batted balls of 66.08 ft.), it is clear that coverage and average distance are critical criteria associated with defensive strategy. Nevertheless, the tradeoffs in Fig. 2 indicate that in all cases an improvement in average distance is possible through the strategic repositioning of outfielders. Average distance to a batted ball can improve by 0.36 ft. up to 2.23 ft., depending on outfielder configuration. However, this can be done in a manner that either increases the percent of demand covered or has minimal impact on coverage. The tradeoff solution in Fig. 5 (coverage of 87.59% and an average distance of 64.29) demonstrates that positioning of outfielders improves average distance by 1.79 ft. (2.71%) while increasing coverage within the 90 ft. standard by 1.81%. This is precisely why the actual fielder positions in Fig. 1 are considered a dominated solution in multiple objective optimization, because there exist position configurations capable of improving both objectives. As a result, this solution is depicted in the interior of the non-dominated solutions in Fig. 2.

## Discussion

The tradeoff possibilities summarized in Fig. 2 highlight that a defensive advantage can be gained through the strategic placement of fielders. An increase in coverage translates to a greater likelihood of a fielder making a catch before the batted ball touches the ground. A decrease in average distance to a batted ball means faster response by a fielder, increasing the changes of making a catch and/or limiting an extra base(s) associated with a hit, but also increasing the potential of throwing someone out while they are advancing bases. Both coverage and average distance increases, no matter how small, translate to a competitive defensive advantage. The outfielder positioning in Fig. 5 is indeed a prime example of this, where coverage can be improved by almost 2% and average distance reduced by almost 3% over current fielding locations. Further, one might consider the outfielder configuration shown in Fig. 5 to be non-intuitive, where there is a decided shift toward the right-field foul line. This is precisely why it is important to take an analytics perspective, where such insights become possible as the performance improvement to respond to batted ball is very significant. While not reported here in detail, analysis for cases where the outfielder range was reduced to 85 ft. (i.e., $S = 85$) as well as increased to 95 ft. (i.e., $S = 95$) was also carried out. This involved the solution of an additional 1,280 problem instances. The results are similar to those summarized previously with respect to tradeoffs and spatial configuration of fielders, though with nuanced differences. For example, for $S =$ 85 the average distance range is [63.80, 65.448] while for $S =$ 95 the range is [63.80, 64.614]. This is expected, as the average distance objective is the same in the best case, but the capability to cover does change. The result is that the associated worst-case average distance is slightly different.

**Fig. 7:**
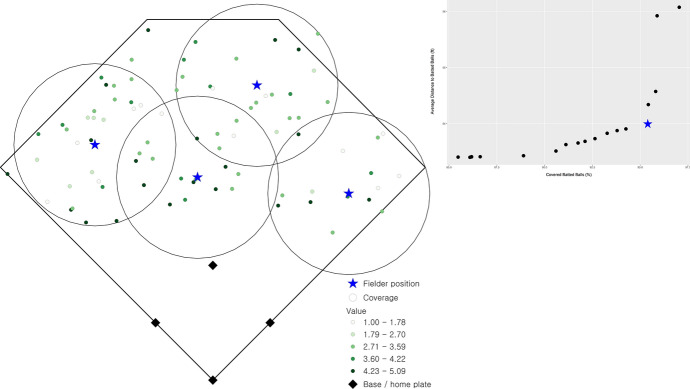
Compromise solution for four fielders (*p* = 4)

There has long been interest in and discussion of the use of an additional outfielder in particular game situations. Recent discussion of this can be found in Miller (2017) and Lindbergh (2018), among others. The idea is that while this makes the infield more challenging to defend, the benefit is increased capability to protect against hits or extra bases for ball batted to the outfield. The proposed modeling framework actually facilitates analysis of why this is beneficial, as well as how to most strategically position players to defend against batted balls. An examination of adding a fourth player to the outfield was carried out. Figure 7 shows the outfield positioning for one case, highlighted in the corresponding inset tradeoff summary (20 non-dominated solutions were identified for this case, $p = 4$). This positioning enables 95.41% coverage of total demand with an average distance to a batted ball of 53.99 ft. by the closest outfielder. The tradeoff configurations range from 84.93% to 97.04% coverage with an associated average distance spanning 53.26 ft. to 56.07 ft. Compared to the three observed outfielder locations in Fig. 1, the positioning of four players in Fig. 7 represents an increase in coverage of nearly 10% and an average distance improvement of over 12 ft. (over 12%). Collectively, the tradeoff solutions for the case of four outfielders demonstrate that average distance to a batted ball can be decreased by more than 10 ft. in the worst case but up to 12.82 ft. in the best case, with all fielder configurations providing enhanced coverage in all but one case. For the best case, an extremely high level of demand coverage (97.04%) is possible. Given the need to prevent a ball to the outfield in certain game situations (e.g., no doubles defense), as well as the propensity for certain batters to hit the ball in the air, there clearly exist measurable advantages to the strategy of placing a fourth fielder in the outfield.

A few discussion items regarding the modeling approach are worth noting. First, the proposed model can be distinguished from those detailed in Church et al. (1991) and Pirkul and Schilling (1991). Both are focused on minimizing average distance only to that demand that is not covered. In our detailed spatial optimization models (1)–(7), there is interest in average distance to field all batted balls. A second point is that various sorts of modeling extensions are possible, such as those detailed in Church and Murray (2009). Examples include backup coverage and secondary response (e.g., Pulver and Wei 2018), where assistance by a second outfielder with respect to coverage or average distance might be explicitly considered. The third point is that the problem of interest here has been viewed in a discrete manner. It was assumed for computational purposes that potential fielder locations were known in advance, enabling a priori derivation of distance between possible positions and batted balls. Indeed, outfielders may be positioned anywhere in the playing field, which reflects an infinite number of potential locations. This is commonly referred to in location modeling as a continuous space problem (see Church and Murray 2009). Continuous space makes this problem even more challenging, with essentially no exact or heuristic solution approaches to solve (1)–(7) at this point in time. This is an open area for further research.

## Conclusions

This paper has detailed the use of a spatial analytic approach to evaluate and optimize fielder positioning. Application in baseball defense was the focus of this initial work, but it is clear that many related sports, like softball, cricket, etc., could readily be addressed using the developed methodology. Baseball and other collegiate and professional sports are big business. Investment in a range of analytic approaches has emerged to support and better understand player performance, situational response, expected behavior, etc. This is invariably done to provide better training, investment, decision making, etc. Ultimately, the intent is to gain strategic advantage in one form or another. The analysis of defense associated with batted balls highlights that current practices for outfielder location do relatively good with respect to both coverage and average distance objectives, but response can be improved in subtle ways through the repositioning of players. This translates to performance improvements of 1% to 3%, depending on the tradeoff configuration selected. Such improvements can be the difference between a game changing play for the defensive team, or not. This is the very essence of strategic planning associated with events that are probabilistic in nature.
